# Evaluating hemodynamic response to treatment in patients with peripheral arterial disease using dynamic vascular optical spectroscopy

**DOI:** 10.1117/1.JBO.29.12.127001

**Published:** 2024-12-24

**Authors:** Nisha Maheshwari, Alessandro Marone, Stephen H. K. Kim, Danielle R. Bajakian, Andreas H. Hielscher

**Affiliations:** a New York University, Tandon School of Engineering, Department of Biomedical Engineering, New York, United States; b Columbia University, Irving Medical Center, Division of Vascular Surgery and Endovascular Intervention, Department of Surgery, New York, United States

**Keywords:** peripheral arterial disease, near-infrared spectroscopy, hemodynamics, peripheral arterial disease management, vascular surgery

## Abstract

**Significance:**

Tracking changes in the vasculature of patients with peripheral arterial disease (PAD) may identify the need for follow-up treatment within only weeks after an initial intervention, enabling timely support and improving patient outcomes.

**Aim:**

We aim to evaluate dynamic vascular optical spectroscopy’s (DVOS’s) ability to accurately monitor the hemodynamics of affected arteries in patients with PAD after a surgical intervention and predict long-term clinical outcomes.

**Approach:**

A DVOS system non-invasively monitored the blood flow through 256 lower extremity arteries in 80 PAD patients immediately before, immediately after, and 3 to 4 weeks after they underwent a surgical intervention.

**Results:**

Hemodynamic changes measured by DVOS after a revascularization procedure (RP) classified patient long-term (6.2±4.4 months) outcomes with high accuracy [81.6% for patients with ulcers (n=31); 81.1% for patients without ulcers (n=54)] by 3 to 4 weeks after the RP, outperforming available ankle-brachial index and ultrasound measurements. In addition, DVOS parameters distinguished between patients who underwent only a catheter angiography (CA) and patients who underwent both a CA and RP (P<0.05).

**Conclusions:**

The DVOS system was able to classify patient long-term clinical outcomes with high accuracy within one month after an RP and distinguish among different interventions. DVOS may be a promising alternative or adjunct to existing monitoring approaches.

## 1 Introduction

Peripheral arterial disease (PAD) is caused by a buildup of plaque in the arteries. In the United States alone, it is estimated that 8 to 12 million people suffer from PAD.^1^ If lifestyle changes cannot sufficiently address PAD symptoms, then surgical intervention is needed. Over 30% of patients require a second surgical intervention within 12 months due to the persistence of symptoms such as ulcers.^2^^,^^3^

The ankle-brachial index (ABI) assessment and arterial duplex ultrasound (DUS) imaging are commonly used to monitor and assess PAD patients after an intervention.^1^^,^^2^^,^^4^ However, both methods have limited use in some patient populations. The ABI assessment has low sensitivity in patients who have arterial calcifications—which are common in diabetics—and was found to be a poor predictor of wound healing for patients with ulcers.^5^^–^^7^ DUS can detect narrowing of vessels in the upper leg but has difficulty visualizing the small blood vessels in the feet.^8^^,^^9^ Vessel narrowing is common in the feet of PAD patients, yet neither ABI nor DUS can provide information about distal perfusion in the feet.^8^^–^^10^

New monitoring techniques have been developed to monitor arterial diseases. To address limitations with diabetic patients, Zhang et al.^11^ developed an artificial neural network model using computed tomography angiography data for predicting the prognosis of diabetic foot ulcers. However, they do not consider PAD patients without ulcers. Cho et al.^12^ developed a machine learning model using data from electronic medical records, which predicts chronic wound healing within 12 weeks (AUC=0.717). The model uses demographic characteristics, patient clinical characteristics, and wound characteristics for prediction but does not account for interventions.^12^ Ruth et al.^13^ tested an implantable wireless sensor in a small animal model and successfully demonstrated the ability to monitor arterial occlusion. They found that continuous, long-term monitoring could detect the reappearance of PAD early and enable timely treatment of the disease. However, this method has yet to be tested in humans.^13^ Golberg et al.^14^ tested laser speckle as a contactless means of classifying diabetic feet in PAD patients and were able to classify healthy and diseased limbs with 90% accuracy. However, they do not differentiate between PAD cases that vary in severity nor do they look at outcome prediction. Many other optical modalities have been used primarily to monitor wound healing in PAD patients, such as near-infrared spectroscopy and optical coherence tomography,^15^ yet gaps exist in research of non-ulcer patients and in predictive studies. There is a need for a new technology that can predict long-term prognosis in PAD patients after surgical intervention for a diverse patient demographic.

Dynamic vascular optical spectroscopy (DVOS) is a non-invasive and non-ionizing technology that has the potential to monitor vascular health in PAD patients. In a previous pilot study with 20 healthy subjects and 20 PAD patients, our group showed that DVOS can correctly differentiate PAD patients from healthy subjects based on differences in blood flow in response to a thigh pressure cuff inflation.^16^^,^^17^ In another pilot study with 14 PAD patients (both diabetic and non-diabetic), we showed that our DVOS system classified long-term wound healing in response to a revascularization procedure with high accuracy, sensitivity (Se), and specificity (Sp).^18^

In this paper, we present the most comprehensive study to date, which includes data from 80 PAD patients, to support the hypotheses that our DVOS system can: (1) monitor changes in blood perfusion in response to a revascularization procedure, (2) assist physicians with making long-term patient prognoses within the first month after a patient’s revascularization procedure, and (3) outperform the existing ABI and DUS methods when used for long-term monitoring.

## 2 Methods

This cross-sectional observational study was approved by the Columbia University Medical Center Institutional Review Board (IRB# AAAK6702) and complies with the Declaration of Helsinki. Informed written consent was obtained for all enrolled patients.

### 2.1 Patient Study Population

A non-random convenience sampling method was used to enroll 95 patients (Table S1 in Supplementary Material) diagnosed with PAD and scheduled for surgical intervention at the Columbia University Irving Medical Center in New York City, New York, United States, between 2016 and 2020. A revascularization procedure was deemed either unnecessary or infeasible for 24 of these patients based on their vasculature. These 24 patients underwent only catheter angiographic imaging. The remaining 71 patients underwent a revascularization procedure in addition to catheter angiographic imaging. The procedure was either balloon-only angioplasty (24 patients), stent angioplasty (40 patients), atherectomy (three patients), or bypass (four patients).

Seven patients were excluded because they had no post-intervention measurements, and eight were excluded because the device detached during the measurements due to the patient’s abrupt movements. The remaining 80 patients were separated into three different patient cohorts, as detailed in Table 1. The cohort of patients who had a revascularization procedure and at least one follow-up (R-FU1) was analyzed for long-term outcomes. The number of follow-up visits for patients in this cohort varied, and 56% of patients had two or more follow-up visits. The last available follow-up occurred an average of 6.2±4.4 months after a patient’s intervention. Patient long-term outcome was determined using metrics from each patient’s last available follow-up visit. Patients who initially had an ulcer(s) were considered to have a positive outcome if they no longer had an ulcer(s) or had a reduction in the size of their ulcer(s) as measured at their last available follow-up. The long-term outcome for patients without ulcer(s) was determined using patient and physician reports (see section 1 in the Supplementary Material). The relationship between patient follow-up retention and long-term outcomes is detailed in Sec. 4.4.

**Table 1:** Cohorts of interest in this paper and the sections in which they are discussed. Sample size (N) is given as the number of patients, not the number of angiosomes.

Cohort name	Description	Location(s) in the paper
CA-POST (N=20)	Patients who had only a catheter angiography (no revascularization procedure) and have available data that were obtained immediately after the intervention.	Sections 3.1 and 4.1
R-POST (N=60)	Patients who had a revascularization procedure (in addition to a catheter angiography) and have available data that were obtained immediately after the intervention.	Sections 3.1 and 4.1
R-FU1 (N=39)	A subgroup of R-POST consisting of patients that had available follow-up (FU1) data 3 to 4 weeks after their revascularization procedure and no prior revascularization procedure within six (6) months before their participation in the study (SM 1.2).	Sections 3.2, 3.3.2, 4.2, and 4.3

### 2.2 Dynamic Vascular Optical Spectroscopy (DVOS) System and Data

**Fig. 1:**
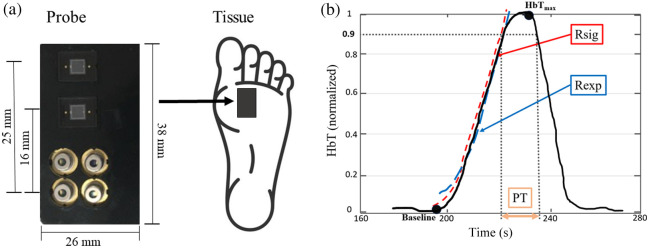
(a) DVOS system’s probe has four sources (670, 708, 808, and 850 nm) and two silicon detectors that collect the reflected light intensity over time of a tissue region of interest. (b) Representative total hemoglobin (HbT) curve showing PT, Rexp, and Rsig parameters. ${\mathrm{HbT}}_{\max}$ indicates the time at which the maximum HbT concentration was reached during the vessel occlusion.

The DVOS system, described in detail in previous publications^16^^–^^19^ and in section 2.1 in the Supplementary Material, was used to assess the blood perfusion of different vascular territories in the lower extremities [Fig. 1(a)]. The DVOS system uses near-infrared spectroscopy to determine the concentration of chromophores of interest in different angiosomes. Each measurement probe [Fig. 1(a); section 2.1 in the Supplementary Material] has four laser diodes with wavelengths in the 670- to 850-nm range. Two silicon photodiodes measure the light reflected through the tissue, and these data are used to calculate chromophore concentrations using a diffusion theory–based algorithm.

This paper analyzes data from three time points, labeled PRE, POST, and FU1. The pre-intervention (PRE) data were acquired 1 to 3 h before the intervention, the post-intervention (POST) data were acquired 1 to 3 h after the intervention, and the first follow-up (FU1) data were acquired 3 to 4 weeks after the intervention. During the PRE data acquisition, up to four measurement probes were placed on the affected leg of each patient. The placement location was maintained across all time points for each patient.

Each probe measures changes in reflected light intensity over time in the tissue under the probe in response to a 60 mmHg (only venous occlusion) and 100 mmHg (venous occlusion and partial arterial occlusion) thigh pressure cuff inflation. The system uses these measured changes to generate so-called total hemoglobin (HbT) concentration curves, which show changes in HbT over time in vascular regions of interest. See section 2.2 in the Supplementary Material for a detailed description of the acquisition procedure.

We extract parameters related to blood perfusion in the angiosomes of interest from these HbT curves [Fig. 1(b)]. In a previous study focusing on PAD patients with ulcers,^18^ we found that the parameter plateau time (PT) was strongly correlated with vascular health. The PT is related to how long it takes for blood to pool and reach saturation after a pressure cuff inflation. A larger PT indicates a healthier vessel, as the blood flows freely without obstruction and the angiosome reaches the maximum hemoglobin concentration shortly after an occlusion is induced, resulting in the concentration plateauing until the pressure cuff is released. A smaller PT occurs when more time is taken to reach the maximum concentration, as the blood must move through partially obstructed vessels.

We introduced two additional parameters in our analysis to account for the fact that patients without ulcers also participated in this study: R-squared exponential fit (Rexp) and R-squared sigmoid fit (Rsig) [Fig. 1(b)]. The Rexp and Rsig parameters are directly related to PT and are measurements of how well exponential and sigmoid functions, respectively, can approximate the shape of an HbT curve. The shape of the curve more closely resembles an exponential or sigmoid function in healthier vessels, where the maximum hemoglobin concentration is reached quickly and the concentration plateaus before the pressure cuff is released. See section 2.3 in the Supplementary Material for details on how each parameter is calculated.

In this paper, we look at the changes between time points of these three parameters for the 60- and 100-mmHg pressure cuff inflations. For example, the differences in PT, Rexp, and Rsig between the PRE and POST time points during the 60-mmHg cuff inflation are stored in the POST-PRE-60 dataset. The same naming convention is used for all datasets: [2nd Time Point]-[1st Time Point]-[Cuff Inflation Pressure].

### 2.3 Ankle-Brachial Index (ABI) and Arterial Duplex Ultrasound (DUS) Methodology

At the FU1 time point, there were 28 patients with available ABI data and 28 patients with available DUS data from the R-FU1 cohort (23 patients had both ABI and DUS data available). These data were taken using the standard medical procedures for ABI and DUS measurements respectively.^5^^,^^20^^–^^22^

The pressure ratio (PR) was considered for the ABI data, and the peak systolic velocity ratio (VR) was considered for the DUS data. Waveforms corresponding to the artery of interest were also considered for the DUS data. Patient outcome was classified using the standard clinical cutoff values for PR and VR + waveforms, detailed in section 3 in the Supplementary Material.

### 2.4 Statistical Analyses

All datasets used for analysis contain data from all the measurement probes (up to four) used on each patient. To account for the inter-dependence of blood perfusion measurements from different locations on the same patient, we computed effective sample sizes (ESS) for all statistical analyses (see section 4 in the Supplementary Material). Table 2 indicates the total sample sizes for each cohort that was analyzed, including the number of patients, the number of arteries, and the ESS.

**Table 2:** Cohorts analyzed and the number of patients and angiosomes for each cohort, as well as the calculated ESS. The ESS is used for statistical analysis.

Dataset	Cohort	Patients	Angiosomes	ESS
POST-PRE-60	CA-POST	20	109	31
	R-POST	80	209	85
POST-PRE-100	CA-POST	20	87	37
	R-POST	80	191	103
FU1-POST-60	Ulcer(s)	12	38	31
	No ulcer(s)	24	90	54
	ABI	26	98	77
	DUS	25	92	69
FU1-POST-100	Ulcer(s)	13	45	29
	No ulcer(s)	24	89	57

All test statistics were adjusted according to the Bonferroni correction protocol. The reported p-values are the effective p-values ($P^{\prime }$) such that $P^{\prime }=P\times N$, where P is the p-value if only one parameter was considered, and N is the number of independent parameters analyzed. Data were considered statistically significant when $P^{\prime }<0.05$.

RStudio (RStudio release 1.4.1717; RStudio, Boston, Massachusetts, United States) was used for the independent samples t-tests in Secs. 3.1, 3.2, and 3.3.2. These data are reported as the mean ± standard deviation.

Quadratic discriminant analysis (QDA) using the pre-defined MATLAB (MATLAB 2021; The MathWorks Inc., Natick, Massachusetts, United States) protocol was performed in Sec. 3.2. We used a two-parameter model with empirical class prior probabilities and no score transform. The model was then cross-validated (CV) over 65 trials using a 10-fold CV method. The accuracy, Se, Sp, and area under the curve (AUC) for the DVOS, ABI, and AD parameters were determined using receiver operating characteristic (ROC) curves analyzed in MATLAB and updated based on the CV results.

The type I (α) and type II (β) error rates of the t-tests and ROC curve analyses were found using the MedCalc (MedCalc release 20.218; MedCalc Software Ltd., Ostend, Belgium) software.

A Spearman’s rank-order correlation test was performed in RStudio to determine the linear dependencies between clinical demographic information, including age, race, claudication status, presence of ulcers, and patient outcome, and PT, Rexp, and Rsig values from all datasets associated with the R-FU1 cohort. Two parameters were considered highly correlated if the correlation coefficient is R>0.7.

## 3 Results

### 3.1 Effects of Revascularization Procedure on Blood Perfusion

The change in PT from the PRE to POST time points was compared between patients with (R-POST cohort) and without (CA-POST cohort) a revascularization procedure for both the 60- and 100-mmHg pressure cuff inflations.

The PT values from the POST-PRE-60 dataset were PT=10.0±13.2  s and PT=0.3±8.4  s for the R-POST (n=85) and CA-POST (n=31) cohorts, respectively. The PT values from the POST-PRE-100 dataset were PT=6.2±9.4  s and PT=1.1±7.6  s for the R-POST (n=103) and CA-POST (n=37) cohorts, respectively.

**Fig. 2:**
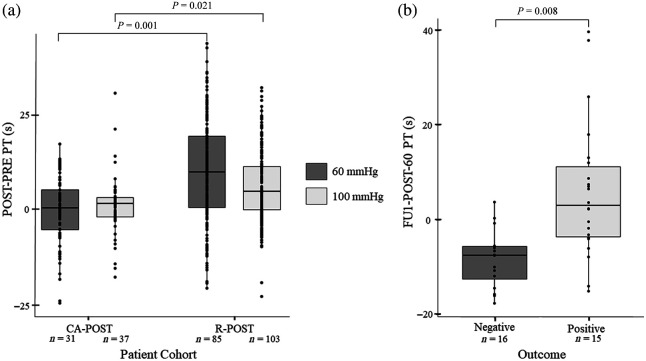
(a) Comparison of PT in the POST-PRE-60 (dark grey) and POST-PRE-100 (light grey) datasets between the CA-POST and R-POST cohorts. Based on the independent samples t-test, the differences between the CA-POST and R-POST cohorts are significant for the POST-PRE-60 dataset (P=0.001) and for the POST-PRE-100 dataset (P=0.021). (b) Comparison of PT in the FU1-POST-60 dataset between patients with ulcers in the R-FU1 cohort with a positive long-term outcome and those with a negative long-term outcome (P=0.008, independent samples t-test).

The PT values were significantly different between the two cohorts for both the POST-PRE-60 (P=0.001, α=0.05, β=0.01) and POST-PRE-100 (P=0.021, α=0.05, β=0.1) datasets [Fig. 2(a)].

### 3.2 Effects of Revascularization on Long-Term Patient Outcome

We found that the DVOS parameters were better able to predict patient outcomes when patients in the R-FU1 cohort were grouped based on whether they had an ulcer(s) at the PRE time point. Analysis of the entire R-FU1 cohort can be found in Table S2 in the Supplementary Material.

**Fig. 3:**
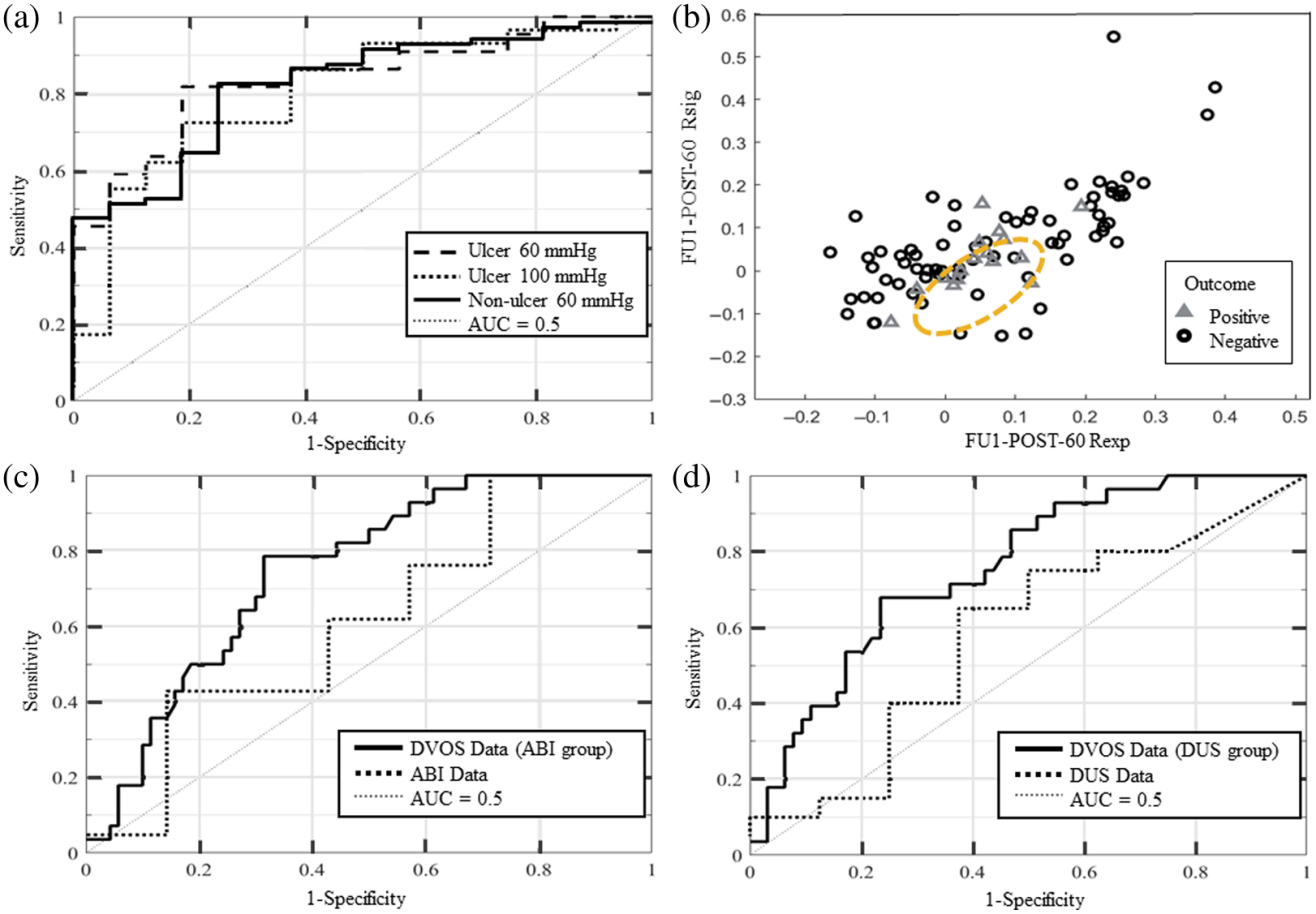
(a) ROC curves for patients in the R-FU1 cohort without ulcers (solid line) using PT cutoff values from the FU1-POST-60 dataset and patients in the R-FU1 cohort with ulcers using PT cutoff values from both the FU1-POST-60 (dashed line) and FU1-POST-100 (dotted line) datasets. The grey dotted line is AUC = 0.5. (b) Rsig versus Rexp from the FU1-POST-60 dataset for patients in the R-FU1 cohort without ulcers who had a negative (black circles) and positive (grey triangles) long-term outcome. The best fit QDA model (orange dashed circle) indicates that patients with a positive outcome have small positive Rexp and Rsig measurements. (c) ROC curves for patients in the R-FU1 cohort with available ABI data using PT cutoff values from the FU1-POST-60 dataset (solid line) and for the same patients using ABI measurements for cutoff values (dotted line). The grey dotted line is AUC = 0.5. (d) ROC curves for patients in the R-FU1 cohort with available DUS data using PT cutoff values from the FU1-POST-60 dataset (solid line) and for the same patients using DUS measurements for cutoff values (dotted line). The grey dotted line is AUC = 0.5.

PT was found to be a good way to differentiate between patients with ulcers that had positive and negative outcomes after a revascularization procedure. For the FU1-POST-60 dataset ($n_{\text{positive}}=15$, $n_{\text{negative}}=16$) patients who had a positive outcome had PT=5.8±14.5  s, which significantly differed (P=0.008, α=0.05, β=0.5) from patients who had a negative outcome and PT=−8.3±6.1 [Fig. 2(b)]. ROC analysis of the FU1-POST-60 dataset differentiated between patient outcomes with accuracy = 81.6%, Se = 81.8%, Sp = 81.3%, and AUC = 0.84 [α=0.05, β=0.2; Fig. 3(a)].

Analysis of the FU1-POST-100 dataset ($n_{\text{positive}}=21$, $n_{\text{negative}}=8$) indicated that there was no significant difference in PT values between patients based on the outcome. However, ROC analysis of the FU1-POST-100 dataset had high accuracy, Se, Sp, and AUC [see Table S2 in the Supplementary Material; Fig. 3(a)].

Single variable analysis was not sufficient to differentiate PAD patients without ulcers based on long-term outcome. QDA was applied to the FU1-POST-60 and FU1-POST-100 datasets for patients without ulcers. Rexp and Rsig were found to be the parameter pair that best classified patients without ulcers based on long-term outcomes [Fig. 3(b)]. Analysis of the FU1-POST-60 dataset ($n_{\text{positive}}=40$, $n_{\text{negative}}=14$) indicated that these data could be differentiated based on the patient long-term outcome with high accuracy =81.1%, Se = 82.4%, Sp = 75%, and AUC = 0.74 [α=0.05, β=0.2; Fig. 3(a)]. The use of the Rexp and Rsig parameters from the FU1-POST-100 dataset ($n_{\text{positive}}=48$, $n_{\text{negative}}=9$) to differentiate patients based on outcome had low accuracy, Se, and Sp (Table S2 in the Supplementary Material).

### 3.3 Comparison of ABI, DUS, and DVOS Data

#### 3.3.1 ABI & AD analysis

Some patients in the R-FU1 cohort had available ABI ($N_{\text{positive}}=21$, $N_{\text{negative}}=7$) and/or DUS ($N_{\text{positive}}=20$, $N_{\text{negative}}=8$) data at the FU1 time point (Table 3). The ABI and DUS measurements from these two groups of patients at the FU1 time point were analyzed to determine their accuracies in predicting long-term patient outcomes.

**Table 3:** Truth table comparing the true patient outcome to the outcome predicted by ABI and DUS clinical cutoff parameters. Cells are bolded when the ABI or DUS outcome matches the true outcome.

Patient	True outcome	ABI	DUS
1	0	**0**	**0**
2	1	0	0
3	0	**0**	X
4	1	0	0
5	1	0	X
6	1	0	0
7	1	0	X
8	1	0	0
9	0	**0**	**0**
10	1	0	X
11	0	**0**	**0**
12	1	0	0
13	0	**0**	**0**
14	1	0	**1**
15	1	0	0
16	1	0	0
17	0	**0**	1
18	1	**1**	0
19	1	**1**	0
20	1	0	**1**
21	1	**1**	0
22	1	**1**	0
23	1	**1**	X
24	1	**1**	0
25	1	**1**	0
26	1	**1**	0
27	1	**1**	0
28	0	1	**0**
29	0	X	1
30	0	X	0
31	1	X	0
32	1	X	0
33	1	X	0

Using PR < 0.9 as the cutoff (section 3 in the Supplementary Material), ABI had accuracy = 53.6% with Se = 85.7% and Sp = 42.9% (AUC = 0.62) when predicting patient outcome [Fig. 3(c)]. Using VR = 0 (absent) or VR > 2.4 combined with the observed waveforms—denoted VR + waveforms—as the cutoff (section 3 in the Supplementary Material), DUS had accuracy = 28.6% with Se = 75.0% and Sp = 10.0% (AUC = 0.58) for predicting patient outcome [Fig. 3(d)].

#### 3.3.2 DVOS Analysis

We also looked at the accuracy of PT in predicting long-term patient outcomes for the two groups analyzed in Sec. 3.3.1. PT was found to be the most correlated with patient outcomes compared with Rexp and Rsig.

The FU1-POST-60 dataset for patients with available ABI data ($n_{\text{positive}}=50$, $n_{\text{negative}}=27$) had significantly different PT values between patients with a positive and negative long-term outcome (P<0.001, α=0.05, β=0.1). PAD patients with a positive outcome had PT=−7.4±3.4  s, and patients with a negative outcome had PT=3.5±10.2  s. PT from the FU1-POST-60 dataset was able to differentiate patients based on their outcomes with high accuracy = 71.4%, Se = 78.6%, and Sp = 68.6% [AUC = 0.75, α=0.01, β=0.1; Fig. 3(c)].

The FU1-POST-60 dataset for patients with available DUS data ($n_{\text{positive}}=46$, $n_{\text{negative}}=23$) also had significantly different PT values between patients with a positive and negative long-term outcome (P<0.001, α=0.05, β=0.1). PAD patients with a positive outcome had PT = −9.2 ± 12.4 s, and patients with a negative outcome had PT=1.7±11.2  s. ROC curve analysis [Fig. 3(d)] also showed that the PT parameter from the FU1-POST-60 dataset had 75% accuracy for predicting patients’ long-term outcomes and Se = 70.4% and Sp = 76.9% (AUC = 0.77, α=0.01, β=0.1).

The FU1-POST-100 group had no significant differences in any of the DVOS parameters between patients with different long-term outcomes in either the ABI or DUS subgroups.

## 4 Discussion

### 4.1 Effects of Revascularization Procedure on Blood Perfusion

We looked at the difference between the PRE and POST time points when comparing the R-POST and CA-POST cohorts because most of the CA-POST cohort did not have available FU1 data. There were significant differences between these two cohorts in the POST-PRE-60 (P=0.001) and POST-PRE-100 (P=0.021) datasets [Fig. 2(a)].

The mean PT values for both cohorts and both datasets were positive, indicating that vascular health improved immediately for all patients regardless of intervention type. The R-POST cohort showed larger improvements in vascular health than the CA-POST cohort across both datasets. The improvement noted in the CA-POST cohort may be due to the catheter and/or contrast agent affecting the plaque stuck to artery walls, which may impact the blood perfusion. These findings suggest that the DVOS system may be able to assess the success of an intervention only 1 to 3 h after the intervention.

### 4.2 Effects of Revascularization on Long-Term Patient Outcome

Of the R-FU1 cohort, 44% of patients were assigned female at birth, 51% were non-white, there was an age range of 48 to 90 years of age, 56% of patients were overweight or obese, 59% were diabetic, and 59% were smokers. The PT, Rexp, and Rsig parameters associated with the R-FU1 cohort were not correlated with sex assigned at birth, race, age, body mass index, diabetic status, or smoking status. This suggests that DVOS is suitable for monitoring the progression of PAD in many different patient populations. A weak correlation was found between patient outcome and diabetic status (R=−0.45) likely because 35% of diabetic patients had a negative outcome, as opposed to 6% of non-diabetic patients. This indicates that the revascularization interventions performed in this study have lower efficacy in patients with diabetes.^4^

When the ulcer and non-ulcer groups were analyzed separately, DVOS parameters were better able to characterize patients based on long-term outcomes. The PT parameter was sufficient for classifying patients based on long-term outcomes in the FU1-POST-60 ulcer subgroup analyzed in this study but was insufficient for classifying the non-ulcer subgroup and the entire R-FU1 cohort. QDA indicated that Rexp and Rsig were the best parameter pair for predicting patient outcomes for both the FU1-POST-60 non-ulcer subgroup (Fig. 3) and the entire R-FU1 cohort (Table S2 in the Supplementary Material). Classification of non-ulcer patients based on a non-linear combination of Rexp and Rsig properties (decision boundaries shown in Fig. 3) may be sufficient for patient long-term outcomes.

The FU1-POST-100 datasets were not sufficient for distinguishing between patients with positive and negative outcomes for patients with and without ulcers. A partial arterial occlusion was achieved for most patients during the 100-mmHg trial, whereas the effects of the 60-mmHg trial, which only achieved a venous occlusion, were likely to be strongly influenced by patient biomarkers such as blood pressure, heart rate, and overall vascular health. This suggests that biomarkers other than hemoglobin concentration may impact the DVOS measurements and the ability to distinguish between patient groups.

FU1-POST-60 PT values had opposite trends for ulcer and non-ulcer patients based on the outcome. We expect patients with a positive outcome to have a positive, large PT value, as PT improves from the POST to FU1 time point. This was true for patients in the R-FU1 cohort with ulcers. However, patients without ulcers in the R-FU1 cohort displayed the opposite trend (negative PT for patients with a positive outcome and positive PT for patients with a negative outcome). The opposing trends may be due to differences in initial vascular health. Patients with and without ulcers are generally in different stages of vascular health, and blood pooling characteristics differ between these groups.^4^^,^^23^^–^^25^ Continued improvements post-intervention may be less pronounced in non-ulcer patients than patients with severe PAD. For patients without ulcers, those who had a negative outcome likely had worse initial vascular health. Small changes in blood perfusion post-intervention in this group could yield a positive PT value, even if the patient does not improve overall.

This study suggests that DVOS may aid physicians in assessing long-term outcomes 3 to 4 weeks after an intervention when different metrics are used for patients with and without ulcers.

### 4.3 Comparison of ABI, DUS, and DVOS Data

ROC curve analysis indicated that ABI and DUS data at the FU1 time point had low AUCs and accuracy when characterizing R-FU1 patients based on long-term outcomes [Figs. 3(c) and 3(d)].

The ABI is known to have low accuracy in diabetic patients, which made up 57% of the R-FU1 cohort with available ABI data. These patients have a high likelihood of arterial calcification, which can skew the PR acquisition.^5^^–^^7^

The low accuracy of the DUS measurements may be due to difficulties in measuring smaller arteries. In these cases, the use of waveforms to determine highly localized vascular health may decrease the accuracy of the monitoring technique.

The PT parameter derived from the optical data of the two groups with available ABI and DUS measurements was also analyzed to directly compare the three monitoring systems. ROC curve analysis of the FU1-POST-60 PT data showed that the PT parameter was more robust than both the PR and VR + waveforms parameters (Sec. 3.3.2). We observed the same trend in the ABI and DUS sub-cohorts as in the non-ulcer R-FU1 cohort, in which patients with a positive outcome had negative PT values and vice versa. Of the ABI and DUS cohorts, only 31% and 28% of patients had ulcers, respectively. Given that the majority of these patients did not have ulcers, it follows that we expect to see the same trend as we do in the non-ulcer R-FU1 cohort.

### 4.4 Limitations of the Study

Determining the true outcomes for non-ulcer patients was a major limitation of this study. In ulcer patients, changes in vascular health are directly related to the change in the size of their ulcer(s), which can be measured quantitatively over time.^4^^,^^24^^,^^25^ However, there were no objective biomarkers or visible wounds for the non-ulcer subgroup, so the outcome was determined with the patients’ self-reports. This could explain the differences between patients with and without ulcers in the ROC curve analysis (Table S2 in the Supplementary Material).

Patient follow-up retention was a further limitation of this study as the number of patient follow ups was not standardized (44% of patients had only one follow up). The amount of time between the intervention and determining the long-term outcome differed for each patient based on the number of follow ups the patient attended. Long-term outcome is more likely to be accurate for patients with ulcers, as visual inspection of ulcer size can determine response to intervention and serve as a visual cue for patients to follow up. Non-ulcer patients had fewer follow ups on average, which may account for the lower classification accuracy, Sp, and AUC as compared with the classification of ulcer patients. The subjectivity of non-ulcer patient outcomes suggests that it may be beneficial to use DVOS to monitor all non-ulcer patients for at least 6 months after an intervention.

## 5 Conclusions

The parameters derived from the DVOS HbT concentration curves can predict patient long-term outcomes 1 month after an intervention with high accuracy (for patients with ulcers, accuracy = 81.6%; for patients without ulcers, accuracy = 81.1%). Our DVOS system had a much higher accuracy in predicting patient outcomes than ABI- and DUS-derived parameters. In addition, the DVOS system detected the effects of a revascularization procedure on blood perfusion in the angiosomes only 1 to 3 h after an intervention.

DVOS has the potential to aid physicians in developing a patient’s treatment plan shortly after an intervention. This may help decrease the duration of a patient’s pain and discomfort and improve their chances of avoiding amputation or a second intervention.

## Supplementary Material



## Data Availability

The datasets presented can be made available for non-commercial reasons upon reasonable request to the author, after acceptable completion of the university’s data use agreement. Requests to access the datasets should be directed to ndm9911@nyu.edu.
