# Supplementary material for: Evaluating hemodynamic response to treatment in patients with peripheral arterial disease using dynamic vascular optical spectroscopy
Source: J Biomed Opt. 2024 Dec 24;29(12):127001. doi: 10.1117/1.JBO.29.12.127001 (PMC11667202; doi:10.1117/1.JBO.29.12.127001)
Supplement: Supplementary file 1 [file JBO_029_127001_SD001.docx]

**Supplementary Materials**

Corresponding paper titled: Evaluating Hemodynamic Response to Treatment in Patients with Peripheral Arterial Disease Using Dynamic Vascular Optical Spectroscopy

Intended for publication as an online data supplement**Supplemental Methods**

**1 Definition of Long-Term Outcome**

The long-term outcomes of the R-FU1 cohort (N=39) were determined in one of two ways. For patients that were included in the ulcer group (N=15), the outcome was determined using information about the ulcer’s size at the patient’s last follow-up with the physician. The patients with a positive outcome had an ulcer(s) that fully healed or reduced in size relative to the PRE assessment. The patients with a negative outcome had an ulcer(s) that did not reduce in size or became larger. For patients that were included in the non-ulcer group (N=24), the outcome was determined using a combination of the patients’ self-report of pain, discomfort, and ability to walk and the monitoring physician’s notes at their last follow-up visit. Physician notes included active problems, presence or resolution of claudication, constitutional review of systems, and a physical exam. The patients with a positive outcome reported reduced pain and/or discomfort and/or greater ease of walking relative to the PRE visit. If a patient reported that there were no improvements in any of these three categories and/or one of them got worse, the outcome was considered negative.

**2 Dynamic Vascular Optical Spectroscopy (DVOS) System**

*2.1 Technology*

The DVOS system supports up to four probes that can simultaneously record localized hemodynamic information from different angiosomes. The probes have four laser diodes that operate in the red and near-infrared range and two silicon photodetectors [Fig. 1(a)] that provide information on the oxygenated and deoxygenated hemoglobin concentrations in the underlying tissue. Data are acquired at the same rate for all probes. More technical details can be found in references 16-19.

The DVOS system is controlled with a laptop that has a custom MATLAB graphical user interface (GUI) that displays, post-processes, analyzes, and stores patient data [Fig. 1(A)]. During data acquisition, the processed signals from all eight source-detector pairs for each probe in use are displayed in real-time on the GUI. In previous publications, we have shown how processed signals are converted into hemodynamic curves using a diffusion-theory based PDE-constrained multispectral reconstruction algorithm.^16^

The signals acquired by the source-detector pairs at each angiosome are processed and sent to the MATLAB GUI (see Section 2.2.1) which extracts the concentration of oxygenated (HbO_2_) and deoxygenated (Hb) hemoglobin over time for each probe. In previous papers, we found the total hemoglobin (HbT), which is the sum of HbO_2_ and Hb, is a potential indicator of vascular health.^18-19^ The GUI displays the changes in HbT concentration over time (as a so-called “HbT curve” [see Fig. 1(B)]) for each probe.

*2.2 Data Acquisition*

Probes were secured to different angiosomes of interest on the affected leg with Velcro straps or 3M Tegaderm transparent film. The affected leg was the leg that was targeted by the surgical intervention. The probe locations were chosen based on ulcer location (if applicable) and physician feedback. Arteries on or nearest to the most affected vasculature were prioritized when placing the probes. Each probe location was noted during the PRE time point and these notes were referenced in the POST and FU1 sessions to ensure that probes remained in the same location ($\pm$10 mm) across time points. Maintaining the probe locations across time points allowed for comparison of localized changes in vascular health over time.

Before any measurements were taken at each time point, the patients were asked about their levels of pain, discomfort, and ease of walking. In addition, any cardiovascular issues (i.e., ulcers, etc.) were noted. Prior to data acquisition, the DVOS system’s gain settings were calibrated for each probe at their respective measurement locations. This allowed the DVOS system to be optimized for a diverse patient demographic. A manual pressure cuff was placed just above the knee, on the thigh of the affected leg. The acquisition process itself consisted of five phases. In the first phase, baseline light intensity values were acquired for one minute. The second phase consisted of a pressure cuff inflation to 60 mmHg which took 8-10 seconds. The cuff was held at this pressure for the remaining part of one minute. In the third phase, the pressure cuff was rapidly deflated in approximately five seconds, and data were acquired while the pump was kept deflated for the remaining part of one minute. In the fourth phase, the pressure cuff was again inflated to 100 mmHg, which took approximately 10-12 seconds. This pressure was maintained for the remainder of one minute. In the final phase, the cuff was rapidly deflated in approximately five (5) seconds and kept deflated for the remaining part of one minute. The cuff inflations caused a venous occlusion and had a minimal (60 mmHg) or partial (100 mmHg) effect on the arteries. This led to an accumulation of blood in the foot, which is measured by the DVOS system as a reduction in the light detected by the source-detectors pairs.

*2.3 Parameters of Interest*

This paper examines three parameters of interest derived from the HbT concentration curves generated by the DVOS system. These parameters are the plateau time (PT), the R-squared exponential fit (Rexp), and the R-squared sigmoid fit (Rsig) [Fig. 1(B)].

The PT of an HbT concentration curve is given in seconds and calculated from (1):

$PT=\left| t_{0.9{HbT}_{max},inflated}-t_{0.9{HbT}_{max},deflated} \right|$ , (1)

where *HbT_max_* is the maximum HbT value of the concentration curve and generally marks the point of pressure cuff release,$t_{0.9{HbT}_{max},inflated}$ is the time point during the pressure cuff inflation at which the HbT value is 90% of *HbT_max_*, and $t_{0.9{HbT}_{max},deflated}$ is the time point after the pressure cuff inflation at which the HbT value is 90% of *HbT_max_*.

To find Rexp and Rsig, exponential and sigmoid functions are first used to approximate the shape of an HbT curve from the baseline value to *HbT_max_*. These functions are determined using an available MATLAB (MATLAB 2021a; The MathWorks Inc., Natick, MA, USA) algorithm. The Rexp and Rsig are then determined based on how well the respective exponential and sigmoid functions fit the data.

**3 Ankle-Brachial Index (ABI) and Arterial Duplex Ultrasound (DUS) Methodology**

The popliteal, posterior tibial, and dorsalis pedis arteries were measured with ABI. The proximal femoral, superficial femoral, popliteal, peroneal, posterior tibial, and anterior tibial arteries were measured for DUS.

The artery of interest was determined in one of two ways. For patients with at least one ulcer, the artery of interest was the artery that shared an angiosome with the ulcer. If it was not possible to get systolic blood pressure (SBP) or peak systolic velocity (PSV) measurements from that artery due to the size of the ulcer, the measurement used was based on the mean SBP or PSV of all arteries immediately following that artery in the arterial tree. In the case where no measurements from the following arteries were available, the measurement used was based on the mean SBP or PSV of all arteries immediately preceding the artery corresponding to the ulcer in the arterial tree. For patients with more than one ulcer, the artery of interest was determined using the same protocol and considered the average of the SBP and PSV most closely related to each ulcer. For patients with no ulcers, the artery of interest was the artery targeted by the intervention. As with ulcer patients, if it was not possible to get SBP or PSV measurements from that artery directly, first the following arteries were considered and then the preceding arteries as necessary. For patients with more than one intervention location, the artery of interest was calculated using the mean of the SBP or PSV measurements most closely related to each artery targeted by an intervention.

For ABI data, the pressure ratio (PR) was given as the ratio of SBP at the artery of interest to the SBP at the brachial artery. For the DUS data, the velocity ratio (VR) was given as the ratio of PSV in the artery of interest to the PSV in the common femoral artery.

We considered standard clinical cutoff values for ABI and DUS to determine patient classification for long-term outcome. For ABI, if PR $\leq$ 0.9 (or PR $\geq$ 1.4 in the case of some diabetic patients), it is an indication of the presence of PAD,^1,3-5,7,20,23,26-27^ and these patients were classified as having a negative outcome. Patients with PR > 0.9 were classified as having positive outcomes. For DUS, if VR = 0 or VR > 2.4, it is an indication of the presence of PAD.^9, 21-22^ Additionally, DUS classification uses waveforms to further identify PAD in patients.^21-22^ Absent, stenotic, continuous, turbulent, tardus/parvus, and monophasic waveforms are signs of PAD while biphasic and triphasic waveforms are considered healthy. Patients with VR = 0 or VR > 2.4 and/or unhealthy waveforms were classified as having negative outcomes. Patients that did not meet the unhealthy VR and waveform criteria were classified as having positive outcomes.

**4 Statistical Analyses**

The effective sample size (ESS) was calculated as follows:

$ESS= \frac{mk}{1+\rho(m-1)}$ . (2)

In (2), *k* is the number of patients, *m* is the average number of probes per patient, and $\rho$ is the intraclass correlation coefficient (ICC).^26^ The ICC accounts for variation within and between patient measurements for single (3) and multivariable (4) analysis. ICC for single variable analysis is defined as

$\rho=\frac{s_{b}^{2}}{s_{b}^{2}+s_{w}^{2}}$ (3)

where $s_{b}^{2}$ is the variance between patients and $s_{w}^{2}$ is the variance within patients. Similarly, for multivariable analysis, the ICC is defined as

$\rho=\frac{\sum_{i,j} |\sigma_{i,j}^{b}|}{\sum_{i,j} |\sigma_{i,j}^{b}|+\sum_{i,j} |\sigma_{i,j}^{w}|}$ (4)

where $\sigma^{b}$ and $\sigma^{w}$ are the between and within cluster covariance matrices, respectively. An open-source general ICC function based on Ahrens analysis was used to calculate the multivariable ICC in RStudio (RStudio release 1.4.1717; RStudio, Boston, MA, USA).^29-30^ All sample sizes (n) referred to in the results of this paper are the ESS unless otherwise specified.

The maximum possible ESS is equivalent to the total number of measurements from all probes and is achieved when there is no inter-dependence between different probes measuring the same patient. The minimum possible ESS is equivalent to the number of patients per cohort.^28^

**Supplemental Tables**

**Table S1**. Patient study population characteristics for all patients (N=95). Data are presented as N (%) or mean ± standard deviation.

| **Characteristics** | **Total** |
| --- | --- |
| *Patient Characteristics* |  |
| Sex – male | 49 (52) |
| Age – years | 70.2 ± 13.7 |
| BMI^*^ – kg/m^2^ | 27.2 ± 5.3 |
| Leg affected – left | 45 (47) |
| Non-white | 49 (52) |
| *Comorbidity* |  |
| History of smoking (current or past) | 50 (53) |
| Diabetic | 55 (58) |
| Family history of diabetes | 20 (21) |
| Hypertension | 62 (65) |
| Hyperlipidemia | 47 (49) |
| *Presenting Symptoms* |  |
| Claudication | 45 (47) |
| Amputation | 1 (1) |
| *Intervention* |  |
| Balloon-only angioplasty | 24 (34) |
| Angioplasty with stent | 40 (56) |
| Atherectomy | 3 (4) |
| Bypass | 4 (6) |

**^*^**BMI: body mass index

**Table S2.** Summary of ROC curve analysis for the R-FU1 cohort, patients in the R-FU1 cohort with ulcers, and patients in the R-FU1 cohort without ulcers.

| **Group** | **Analysis** | **Pressure** | | **Parameter(s)** | **Accuracy (%)** | **Se (%)** | | **Sp (%)** | **AUC** |
| --- | --- | --- | --- | --- | --- | --- | --- | --- | --- |
| R-FU1 | CV QDA | 60 | Rexp, Rsig | | 75.8 | 71.9 | 77.1 | | 0.75 |
|  |  | 100 | Rexp, PT | | 51.5 | 46 | 68.8 | | 0.58 |
|  |  |  |  | |  |  |  | |  |
|  | ABI | N/A | PR | | 53.6 | 85.7 | 42.9 | | 0.62 |
|  |  |  |  | |  |  |  | |  |
|  | DUS | N/A | VR+waveforms | | 28.6 | 75 | 10 | | 0.58 |
| Ulcers | DVOS | 60 | PT | | 81.6 | 81.8 | 81.3 | | 0.84 |
|  |  | 100 |  |  | 75.6 | 72.4 | 81.3 | | 0.79 |
|  |  |  |  | |  |  |  | |  |
|  | ABI | N/A | PR | | 60 | 66.7 | 57.1 | | 0.56 |
|  |  |  |  | |  |  |  | |  |
|  | DUS | N/A | VR+waveforms | | 45.5 | 100 | 14.3 | | 0.68 |
| No ulcers | CV QDA | 60 | Rexp, Rsig | | 81.1 | 82.4 | 75 | | 0.74 |
|  |  | 100 |  |  | 69.7 | 74.0 | 50 | | 0.61 |
|  |  |  |  | |  |  |  | |  |
|  | ABI | N/A | PR | | 50 | 100 | 35.7 | | 0.62 |
|  |  |  |  | |  |  |  | |  |
|  | DUS | N/A | VR+waveforms | | 23.5 | 75 | 7.7 | | 0.56 |

Se = sensitivity; Sp = specificity; AUC = area under the curve; CV QDA = 10-fold cross validated quadratic discriminant analysis; ABI = ankle-brachial index; DUS = arterial duplex ultrasound; PR = pressure ratio; VR = peak systolic velocity ratio; DVOS = dynamic vascular optical spectroscopy
